# Experiences of Governments and Public Health Agencies Regarding Crisis Communication During the COVID-19 Pandemic in the Digital Age: Protocol for a Systematic Review of Qualitative Studies

**DOI:** 10.2196/58040

**Published:** 2024-06-27

**Authors:** Tsuyoshi Okuhara, Marina Terada, Hiroko Okada, Takahiro Kiuchi

**Affiliations:** 1 Department of Health Communication School of Public Health The University of Tokyo Tokyo Japan; 2 Department of Health Communication Graduate School of Medicine The University of Tokyo Tokyo Japan

**Keywords:** COVID-19, health communication, infodemic, misinformation, social media, SARS-CoV-2, coronavirus, pandemic, infectious, digital age, systematic review, internet, public health, government, governments, crisis communication, qualitative, methodology, disinformation, eHealth, digital health, medical informatics

## Abstract

**Background:**

Governments and public health agencies worldwide experienced difficulties with social media–mediated infodemics on the internet during the COVID-19 pandemic. Existing public health crisis communication strategies need to be updated. However, crisis communication experiences of governments and public health agencies worldwide during the COVID-19 pandemic have not been systematically compiled, necessitating updated crisis communication strategies.

**Objective:**

This systematic review aims to collect and organize the crisis communication experiences of senders (ie, governments and public health agencies) during the COVID-19 pandemic. Our focus is on exploring the difficulties that governments and public health agencies experienced, best practices in crisis communication by governments and public health agencies during the COVID-19 pandemic in times of infodemic, and challenges that should be overcome in future public health crises.

**Methods:**

We plan to begin the literature search on May 1, 2024. We will search PubMed, MEDLINE, CINAHL, PsycINFO, PsycARTICLES, Communication Abstracts, and Web of Science. We will filter our database searches to search from the year 2020 and beyond. We will use a combination of keywords by referring to the SPIDER (Sample, Phenomenon of Interest, Design, Evaluation, and Research type) tool to search the abstracts in databases. We intend to include qualitative studies on crisis communication by governments and public health agencies (eg, officials, staff, health professionals, and researchers) to the public. Quantitative data–based studies will be excluded. Only papers written in English will be included. Data on study characteristics, study aim, participant characteristics, methodology, theoretical framework, object of crisis communication, and key results will be extracted. The methodological quality of eligible studies will be assessed using the Joanna Briggs Institute critical appraisal checklist for qualitative research. A total of 2 independent reviewers will share responsibility for screening publications, data extraction, and quality assessment. Disagreement will be resolved through discussion, and the third reviewer will be consulted, if necessary. The findings will be summarized in a table and a conceptual diagram and synthesized in a descriptive and narrative review.

**Results:**

The results will be systematically integrated and presented in a way that corresponds to our research objectives and interests. We expect the results of this review to be submitted for publication by the end of 2024.

**Conclusions:**

To our knowledge, this will be the first systematic review of the experiences of governments and public health agencies regarding their crisis communication to the public during the COVID-19 pandemic. This review will contribute to the future improvement of the guidelines for crisis communication by governments and public health agencies to the public.

**Trial Registration:**

PROSPERO CRD42024528975; https://tinyurl.com/4fjmd8te

**International Registered Report Identifier (IRRID):**

PRR1-10.2196/58040

## Introduction

The COVID-19 pandemic has been one of the most devastating health crises in human history. The pandemic forced governments and public health agencies to deal with a dizzying array of diverse and complex challenges including disruptions to daily life, economic crises, and rapidly mutating viruses. Governments and public health agencies needed to communicate effectively to the general public to encourage people to restrict their activities and take preventive actions to stop the spread of the virus [1]. Even before the COVID-19 pandemic, the World Health Organization published guidelines for crisis communication [2-4], and public health researchers considered emergency communication strategies based on lessons learned from past public health crises [5-9]. However, once the COVID-19 pandemic broke out, governments and public health agencies worldwide realized that they lacked systematic preparation and training in communicating with the public in the midst of a rapidly changing and confusing situation [10-15].

During the COVID-19 pandemic, governments and public health agencies worldwide experienced great difficulties with crisis communication, represented by an “infodemic” on social media [16]. The flood of information and misinformation made it difficult for people to find reliable sources of information and guidelines for necessary actions to take [17]. For governments and public health agencies, social media was a new and important means of delivering accurate information to the public quickly and widely [18]. At the same time, however, the infodemics allowed misinformation to spread through social media as quickly as the virus [19]. Governments and public health agencies had to fight both the virus and the infodemic. Even before the COVID-19 pandemic, the management of misinformation was recognized as an important public health issue, and many studies were conducted and coping measures proposed [20-23]. However, the COVID-19 pandemic highlighted the inexperience and the failure of governments and public health agencies to develop strategies to deal with the spread of misinformation during an emergency with infodemics [24,25].

Thus, the COVID-19 pandemic presented governments and public health agencies with unprecedented challenges and necessitated updates to existing crisis communication strategies. Our study will collect and organize data on the difficulties experienced and lessons learned by governments and public health agencies worldwide in crisis communication to the general public during the COVID-19 pandemic. This work is essential for updating future crisis communication strategies of governments and public health agencies. To this end, we will conduct a systematic review of qualitative studies on governments and public health agencies in diverse countries. We will focus on the difficulties that governments and public health agencies experienced, best practices of crisis communication from governments and public health agencies to the public during the COVID-19 pandemic in times of infodemic, and challenges to overcome in future public health crises.

## Methods

### Overview

We will conduct and report this systematic review following the guidelines provided in the PRISMA (Preferred Reporting Items for Systematic Reviews and Meta-Analyses) statement (Multimedia Appendix 1) [26]. As this will be a systematic review of qualitative studies, we will refer to the SPIDER (Sample, Phenomenon of Interest, Design, Evaluation, and Research type) tool for the synthesis of qualitative evidence [27]. This protocol is registered with PROSPERO; the registration number is CRD42024528975. We plan to begin the literature search after this protocol is peer-reviewed on May 1, 2024, and finish the analysis by September 30, 2024.

### Literature Search

We will search PubMed, MEDLINE, CINAHL, PsycINFO, PsycARTICLES, Communication Abstracts, and Web of Science. We will filter our database searches to include only papers from the year 2020 and beyond. We will use a combination of keywords with reference to previous studies to search the abstracts in databases [28-30]: ((government*) OR (ministr*) OR (department*) OR (office*) OR (municipalit*) OR (prefecture*) OR (province*) OR (state*) OR (count*) OR (organization*) OR (institution*) OR (center*) OR (agenc*) OR (sector*) OR (authorit*)) AND ((covid-19) OR (coronavirus) OR (sars-cov-2)) AND ((interview*) OR (focus group*) OR (questionnaire*) OR (survey*)) AND ((communicat*) OR (messag*) OR (inform*) OR (recommend*) OR (announce*)) AND ((qualitative) OR (mix method)). All publications collected from the start to the end of the database search will be included. The reference lists of eligible studies will be searched to identify further potentially eligible publications.

### Eligibility Criteria

The inclusion and exclusion criteria for the review are presented in Textbox 1.

**Inclusion and exclusion criteria.**

**Inclusion criteria**

- Qualitative studies of communication to the public by governments and public health agencies
- With regard to design, qualitative studies with qualitative data such as interviews, documents, and free-text responses to the questionnaire
- Content analysis of qualitative data that meets our study aim
- A review of qualitative studies that meet our study aim
- Mixed methods studies with qualitative results that meet our study aim
- With regard to study participants, studies on individuals such as officials, staff, health professionals, and researchers working for governments and public health agencies
- Studies on participants of any age, gender, ethnicity, or country
- Gray literature (information produced outside traditional publishing and distribution channels such as conference proceedings and theses) that provides sufficient information to assess eligibility (ie, full-length description of research objectives, methods, results, discussion, and conclusions)
- Papers written in English
- Studies conducted in January 2020 and beyond

**Exclusion criteria**

- Quantitative studies with quantitative data such as observational and interventional studies
- Studies on journalists in media companies and members of the public
- Studies not published in full-text format
- Papers written in languages other than English
- Studies that do not meet our study aims such as content analysis of media information, studies on information searches by the public, studies on handling patients with COVID-19 in hospitals, patient-provider communication, telehealth, and digital transformation technology

### Study Selection

Rayyan software [31] will be used for screening studies. Duplicates will be removed automatically using this software. First, titles and abstracts will be screened to identify eligible studies using the selection criteria. The titles and abstracts of the literature will be independently screened by both the first (TO) and second (MT) reviewers. Disagreements will be discussed until a consensus is reached. When a consensus cannot be reached, a third reviewer (HO) will be involved in resolving the disagreement. Second, the full text of the remaining literature will be screened independently by both the first (TO) and second (MT) reviewers. If they disagree, the third reviewer will be consulted to resolve it through discussion. The screening process will be displayed using a PRISMA flow diagram.

### Data Extraction

The first reviewer (TO) will extract descriptive data from eligible studies using Microsoft Excel, and the second reviewer (MT) will review the eligible studies for errors in the extracted data. The extracted data will consist of study characteristics (eg, author, year of publication, country, title, year and month of data collection, and type of paper), study aim, participant characteristics (eg, number of participants, workplace, and job title), methodology (eg, study design, methods of analyzing qualitative data, type of data, and setting), theoretical framework (when used), and object of crisis communication (eg, lockdown, behavioral restrictions, preventive behavior, and vaccination). The first reviewer (TO) will extract key results (eg, categories, themes, and quotations) using Microsoft Word, and the second reviewer (MT) will review the eligible studies for errors and bias in the extracted data. The third reviewer (HO) will be consulted if necessary for this validation.

### Quality Assessment

To assess the methodological quality of eligible studies, the first reviewer (TO) will use the Joanna Briggs Institute (JBI) critical appraisal checklist for qualitative research [32], which is a trusted tool used for critical appraisal of qualitative studies [33]. Each of the 10 items on the checklist can be evaluated as “yes,” “no,” “unclear,” or “not applicable.” The JBI checklist assesses the descriptive, interpretative, theoretical, and evaluative validity of qualitative studies. The second reviewer (MT) will validate the results of the quality assessment using the JBI checklist, and the third reviewer will be invited if necessary. Our review will not exclude the included studies because of the results of the quality assessment.

### Data Synthesis

The first reviewer (TO) will synthesize data using thematic synthesis [34]. Thematic synthesis, recommended by Cochrane, is a systematic method for synthesizing qualitative evidence [35]. According to the thematic synthesis method, in the first stage, TO will conduct free line-by-line coding of texts and quotations in the Result section of each of the included studies. In the second stage, the first (TO) and second (MT) reviewers will independently group similar codes generated in the previous stage and develop data-driven descriptive themes. They will reach a consensus through discussion, and the third reviewer (HO) will be consulted if necessary. In the third stage, TO will develop analytical themes by interpreting the descriptive themes generated in the previous stage. Those analytical themes will include new insights beyond the results of individual studies. The process of generating analytical themes will involve discussions between TO, MT, and HO. Those codes and themes will inductively be generated through the data-driven data synthesis process.

## Results

We will summarize our findings in a table and a conceptual diagram and discuss them in a descriptive and narrative review form. We will discuss the implications for future research, policies, and practices. Our findings will be presented at a relevant conference and published in a peer-reviewed journal. We expect our review to be submitted for publication by the end of 2024.

## Discussion

### Expected Findings

Many studies have already been conducted worldwide on the infodemic experienced by patients and citizens during the COVID-19 pandemic [36-44], and systematic reviews of those studies already exist [45,46]. However, no systematic review to date has collected data on the experiences of governments and public health agencies regarding crisis communication during the COVID-19 pandemic. To fill this gap, our systematic review of qualitative studies will collect and organize data on the difficulties and challenges experienced by governments and public health agencies worldwide in crisis communication during the COVID-19 pandemic. Our systematic review will describe how communication strategies were developed and implemented by government and public health agencies during the pandemic, and how they were disrupted, called into question, and changed by the infodemic. It will also reveal outstanding attempts to overcome communication difficulties regarding lockdowns, recommendations for preventive behaviors, and the safety and efficacy of rapidly developed vaccines by governments and public health agencies.

Our systematic review will shed light on the experiences of information senders during the COVID-19 pandemic. By cross-checking studies on the experiences of patients and citizens on the receiving end of information with our systematic review of the experiences of governments and public health agencies on the sending end of information, we will be able to capture a fuller picture of crisis communication experiences during the COVID-19 pandemic. A literature review that provides such a complete picture of crisis communication experiences during the COVID-19 pandemic will be an essential resource for professionals and the public to learn from the COVID-19 pandemic and address future public health crises.

### Limitations

Although we will use a comprehensive search strategy, we may miss relevant studies. To review qualitative studies, such as papers published in international academic journals, we will, as a first step in our research, include full-length literature written in English, which may exclude relevant studies published in languages other than English. Future studies will be expected to collect more comprehensive findings from various countries’ literature written in various languages. This review will include qualitative analyses but exclude quantitative results of previous studies. That is because a quantitative survey using a questionnaire reveals the quantitative distribution of already known or inferred items. Our study objective is to examine the unprecedented difficulties that governments and public health agencies experienced during the pandemic involving the first web-based infodemics in human history, and it attempts to address these difficulties. To this end, it is important to begin with a review of qualitative studies that shed light on the lived experiences of governments and health professionals. We will include analyses of qualitative data, such as interviews, documents, and free-text responses to the questionnaire, to meet our study objective as comprehensively as possible.

### Conclusion

To our knowledge, this will be the first systematic review to describe the experiences of governments and public health agencies around their crisis communication to the public during the COVID-19 pandemic. This review will provide important information for the future improvement of the guidelines for crisis communication of governments and public health agencies to the public. Based on this systematic review, we will examine the communication challenges for governments and public health agencies in the COVID-19 pandemic and provide recommendations for managing future global public health crises.

## Appendix

Multimedia Appendix 1 The PRISMA (Preferred Reporting Items for Systematic Reviews and Meta-Analyses) checklist.

## Abbreviations

JBI: Joanna Briggs Institute
PRISMA: Preferred Reporting Items for Systematic Reviews and Meta-Analyses
SPIDER: Sample, Phenomenon of Interest, Design, Evaluation, and Research type

## Data Availability

Data sharing is not applicable to this article as no data sets were generated or analyzed in this study protocol.
